# Radical Scavenging by Acetone: A New Perspective to Understand Laccase/ABTS Inactivation and to Recover Redox Mediator

**DOI:** 10.3390/molecules201119672

**Published:** 2015-11-04

**Authors:** Hao Liu, Pandeng Zhou, Xing Wu, Jianliang Sun, Shicheng Chen

**Affiliations:** 1 State Key Laboratory of Pulp and Paper Engineering, South China University of Technology, Guangzhou 510640, China; wuxingziyuan12@gmail.com (X.W.); 15083469185@163.com (J.S.); 2 School of Chemistry and Environmental Engineering, Hunan City University, Yiyang 413000, China; PandengZhou2003@yahoo.com; 3 Department of Microbiology and Molecular Genetics, Michigan State University, East Lansing, MI 48824, USA

**Keywords:** laccase, ABTS, inhibition, radical scavenging, recovery

## Abstract

The biosynthetic utilization of laccase/mediator system is problematic because the use of organic cosolvent causes significant inhibition of laccase activity. This work explored how the organic cosolvent impacts on the laccase catalytic capacity towards 2,2′-azino-bis(3-ethylbenzothiazoline-6-sulfonic acid) (ABTS) in aqueous solution. Effects of acetone on the kinetic constants of laccase were determined and the results showed *K_m_* and *V*_max_ varied exponentially with increasing acetone content. Acetone as well as some other cosolvents could transform ABTS radicals into its reductive form. The content of acetone in media significantly affected the radical scavenging rates. Up to 95% of the oxidized ABTS was successfully recovered in 80% (*v*/*v*) acetone in 60 min. This allows ABTS to be recycled at least six times with 70%–75% of active radicals recovered after each cycle. This solvent-based recovery strategy may help improve the economic feasibility of laccase/ABTS system in biosynthesis.

## 1. Introduction

Laccases (EC 1.10.3.2) have been intensively studied as a green catalyst for biosynthesizing polyphenols [1], heterocyclic compounds [2], graft copolymers [3], *etc.* When laccase is applied to oxidize a monomer with a high redox potential (e.g., exceeded 780 mV *vs.* NHE), redox mediators are required for promoting enzyme catalytic efficiency or initiating the oxidation [4]. An ideal mediator should be a recyclable small molecule that is stable in its oxidized or reduced form in reaction media [2]. ABTS (2,2′-azino-bis(3-ethylbenzothiazoline-6-sulfonic acid) is one of the most effective mediator because its radical has a sufficiently high redox potential (e.g., +1100 mV) and is very stable in aqueous solution [5]. However, laccase-catalyzed organic synthesis is preferentially conducted in homogeneous aqueous/organic (A/O) media, *i.e.*, 50% (*v*/*v*) aqueous acetone, in order to dissolve hydrophobic substrates and improve the yield and molecular weight of polymer products [6,7]. In A/O media laccase activity towards ABTS and many other substrates is significantly decreased. The stability and recyclability of either enzyme or mediator are also inevitably influenced by the organic cosolvent.

It remains unclear how the organic solvent impacts on the laccase-ABTS oxidation. Previous studies showed that the variation of medium polarity adversely changed the enzyme conformation and therefore reduced the affinity to substrates [7,8]. There is another possible interpretation that free radicals derived from laccase catalysis could be transformed into their original state in A/O media. However, very few reports are available on the roles of organic solvent in scavenging ABTS radicals. On the other hand, the relatively high cost of ABTS prevents its practical use unless it can be feasibly recovered. The only available process based on ammonium sulfate precipitation [9] is not practical because the subsequent separation is laborious and any residual salt may be harmful to the reuse of ABTS in biosynthesis. In this work we provided a new insight into ABTS radical transformation in A/O media. Thereafter, the feasibility of recovering ABTS using aqueous acetone was discussed.

## 2. Results and Discussion

### 2.1. Inhibition of Laccase-Catalyzed ABTS Oxidation by Acetone

The Michaelis-Menten constants of laccase reacting with ABTS in aqueous/acetone (Aq/Ac) solutions were listed in Table 1. The apparent binding constant, *K_m_*, increased exponentially with increasing acetone content (0%–50%, *v*/*v*). Under the same conditions, the maximum rate of oxidation, *V*_max_, decreased exponentially with the increasing acetone concentration. The *V*_max_ measured in 50% (*v*/*v*) Aq/Ac was two orders of magnitude lower than that in the reference aqueous solution (Table 1). The catalytic efficiency (*V*_max_/*K_m_*) was also exponentially correlated with the acetone contents (Table 1). The *V*_max_/*K_m_* of laccase in 50% Aq/Ac dropped four orders of magnitude compared to the reference test. Kinetics studies of laccase were not determined in a media containing 60% (*v*/*v*) acetone or higher because the initial oxidation velocity was too minimal to be precisely determined and protein precipitation interfered with the test.

**Table 1:** Michaelis-Menten constants of laccase in aqueous media of different acetone contents.

Acetone Content (%, *v*/*v*)	*K_m_* (μM)	*V*_max_ (μM·min^−1^·[E]^−1^ *)	*V*_max_/*K_m_* (L·mg^−1^·min^−1^)
0	7.67 ± 0.44	42.86 ± 0.49	5.588
12.5	25.07 ± 0.65	28.76 ± 0.45	1.147
25	58.66 ± 2.24	8.13 ± 0.24	0.139
37.5	91.99 ± 0.99	4.03 ± 0.08	0.044
50	370.08 ± 20.55	1.94 ± 0.04	0.005

* [E] represents concentration of enzyme, mg·L^−1^.

The effect of acetone on *K_m_* of laccase for ABTS, an ionic substrate, was similar to that reported on *C. unicolor* laccase and neutral substrates such as syringaldazine and 2,6-dimethoxyphenol; whereas, *V*_max_ declined linearly with increasing acetone content in the reference article [10]. Instead, *V*_max_ of *C.* *hirsutus* laccase determined using syringaldazine substrate varied exponentially with acetone concentration, while *K_m_* has a linear relationship with acetone concentration [11]. The difference may be attributed to different laccase sources and/or substrate properties. Moreover, the testing range of solvent contents may have significant impacts on the correlation results. A previous work by Rodakiewicz-Nowak *et al.* indicated that the profile of *V*_max_ towards concentration of water-miscible solvent had been divided into three regions: (1) inhibition region with small decrease in catalytic rate; (2) denaturation region with a dramatic decrease in enzymatic activity; (3) residue region where few enzyme remains active [7]. Thus, the linear inhibition region was not observed possibly due to that the acetone content was too low to inhibit laccase activity considerably, *i.e.*, protein was not denatured. Simple kinetic models like hyperbolic, non-competitive, or uncompetitive were not satisfied for describing the complicated mechanisms of solvent inhibition. Therefore, Rodakiewicz-Nowak *et al.* used a mixed inhibition model integrating binding constants of solvent molecules with both enzyme and enzyme—substrate complex [7].

It should be noted that hydration of enzyme, a prerequisite step for its catalytic action, was related to the water thermodynamic activity [12]. Organic solvents destructed the protein hydration shell resulting in protein denaturation [11]. Solvents also influenced substrate-enzyme binding by altering pH, polarity, and/or hydrophobicity of the media [7,13]. Furthermore, the influence of solvent on intermediate was possibly involved in the catalytic process, *i.e.*, destabilizing intermediate radicals and slowing down the conversion of substrates should be counted [14].

### 2.2. Scavenging ABTS Radicals by Acetone

**Figure 1:**
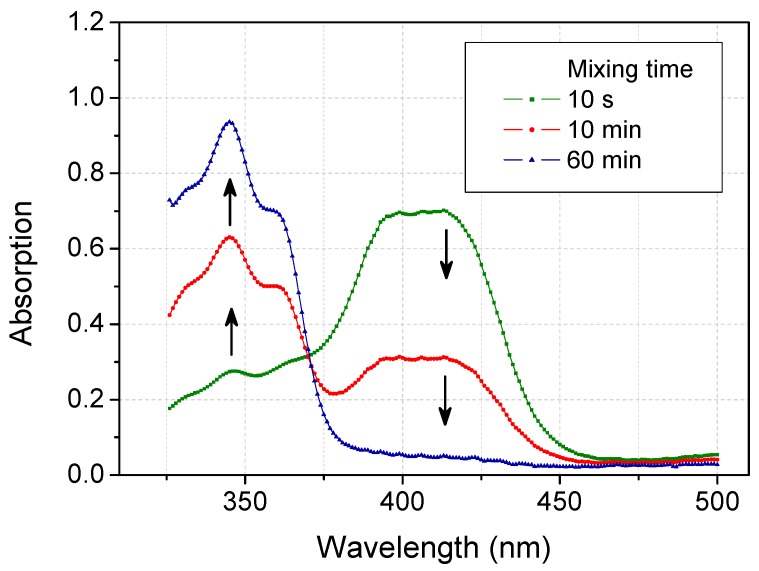
Time-dependent spectra of 2,2′-azino-bis(3-ethylbenzothiazoline-6-sulfonic acid) (ABTS) radicals in 80% (*v*/*v*) Aq/Ac.

We further evaluated the capacity of organic solvent to transform the radicals to the reductive or a new form by a kinetic UV-Vis spectroscopic analysis. Figure 1 showed that ABTS radicals (420 nm) generated by laccase catalysis were nearly transformed into their original state (340 nm) in 80% (*v*/*v*) Aq/Ac in 60 min. In particular, scavenging of ABTS radicals in the initial stage (e.g., in the first 10 min) was much faster than that in the subsequent stage (e.g., in the following 50 min).

**Figure 2:**
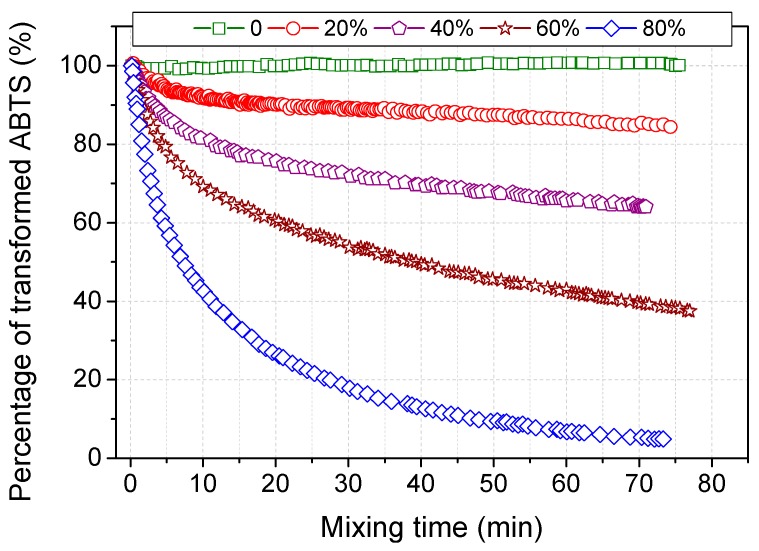
Effects of acetone content on kinetic transformation of ABTS radicals.

**Figure 3:**
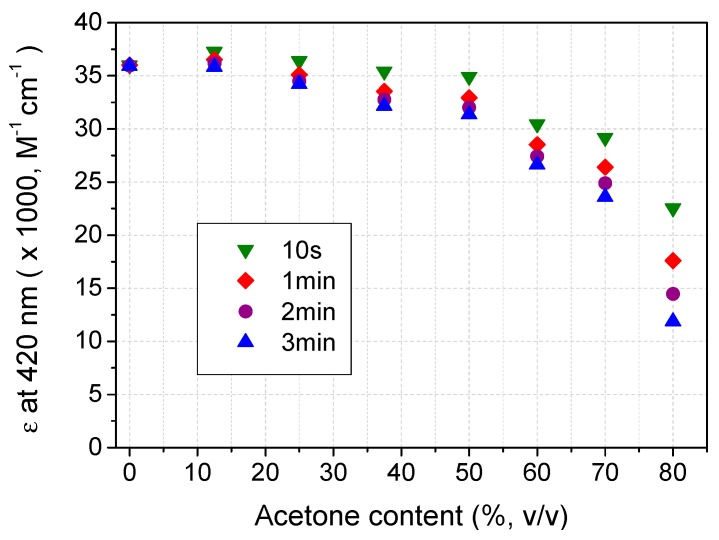
Effects of mixing time on ε of ABTS radicals in Aq/Ac media.

Attenuation of scavenging velocity with time is more clearly shown in Figure 2. Half of ABTS radicals were scavenged in the initial 7 min while 94.5% of radicals were consumed in 60 min. The decreasing velocity was observed in the tested acetone content. The amount of recovered ABTS was greatly dependent on the acetone content, indicating that acetone contributed to the reductive transformation of ABTS radicals. Such transformation results in variation of absorption coefficient of ABTS at 420 nm (ε_420nm_) in Aq/Ac. Acetone contents should be taken into consideration when measuring laccase activity in A/O media. Figure 3 indicates the standard derivation of ε_420nm_ at 1 min was within ±2.5% in the media with 0%–25% acetone, while ±6% was for 25%–50% Aq/Ac.

Transformation of ABTS radicals (ABTS**^·^**^+^) also occurred when other organic solvents were used instead of acetone. ABTS^+^ can be reduced to ABTS by oxidizing a broad spectrum of lignin derived alcohols, aromatic aldehydes, ketones as well as many organic acids (e.g., acetate, glyoxylate, and malonate) [15,16,17]. However, there was no direct evidence showing that acetone or other water-miscible organic solvent can be oxidized by ABTS radicals. Our results showed that ABTS**^·^**^+^ can be scavenged by a variety of water-miscible organic solvents that follows a capacity order of 1,4-dioxane, acetone, isopropanol, ethanol, methanol, and acetic acid (Table 2) indicating that the polarity of organic solvents was a major influence factor. However, the oxidation mechanisms (especially the resulting products) should be further investigated for better understanding the phenomena of solvent-induced radicals scavenging.

**Table 2:** Scavenging of ABTS**^·^**^+^ by various water-miscible organic solvents (80% volume percentage).

Solvents	Reduction Percentage at 45 min (%)	Time for 50% Reduction (min)	Time for 90% Reduction (min)
Acetic acid	9.6 ± 0.6	ND *	ND
Methanol	39.3 ± 1.6	83 ± 2.9	346 ± 4.5
Ethanol	50.5 ± 0.4	41 ± 1.8	209 ± 3.5
Isopropanol	84.5 ± 0.9	15 ± 0.4	51 ± 1.3
Acetone	88.7 ± 1.1	7 ± 0.1	47 ± 0.4
1,4-Dioxane	94.1 ± 0.9	2 ± 0.1	13 ± 0.7

* ND: Not detected.

### 2.3. Recovery of ABTS in Aqueous Acetone Media

**Figure 4:**
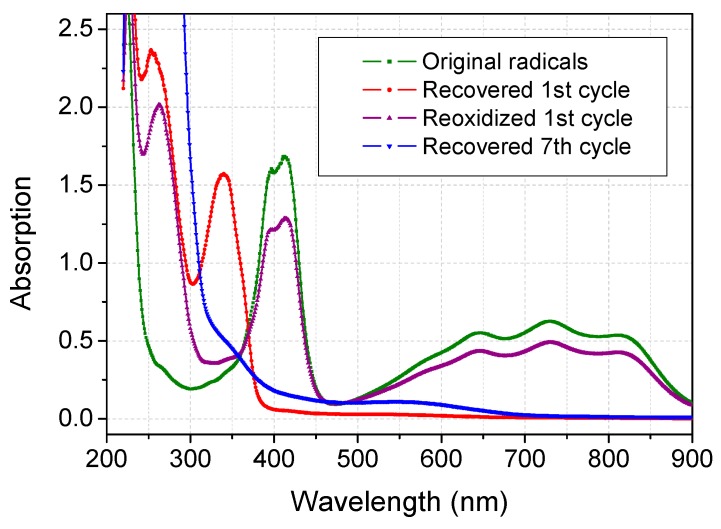
Variation of UV-Vis spectra of recovered ABTS and its radicals.

The capacity of acetone to scavenge ABTS**^·^**^+^ could possibly provide a new strategy to recover ABTS in reductive state (active one). ABTS**^·^**^+^ can couple with some of the reactants or products and longer incubations periods often lead to less recovery [9,18]. UV-Vis spectra of recovered ABTS and its regenerated radicals were demonstrated in Figure 4. Specific absorption at 340 nm of reductive ABTS appeared after the first cycle of recovery, but no specific peak at 420 nm or 730 nm appeared. There was some weak absorbance in 400–700 nm, which correlated with faded ABTS with slight magenta color. This suggests a new form of ABTS was generated. The reaction mixture was turned to be blue-green radicals after acetone was removed by evaporation and re-oxidized by laccase. However, the peak of specific absorbance at 420 nm decreased, *i.e.*, around 20.8% of ABTS cannot be recovered. Our recovery rate was much higher than that using the ammonium sulfate precipitation method (nearly 70% unrecoverable) [9].

**Figure 5:**
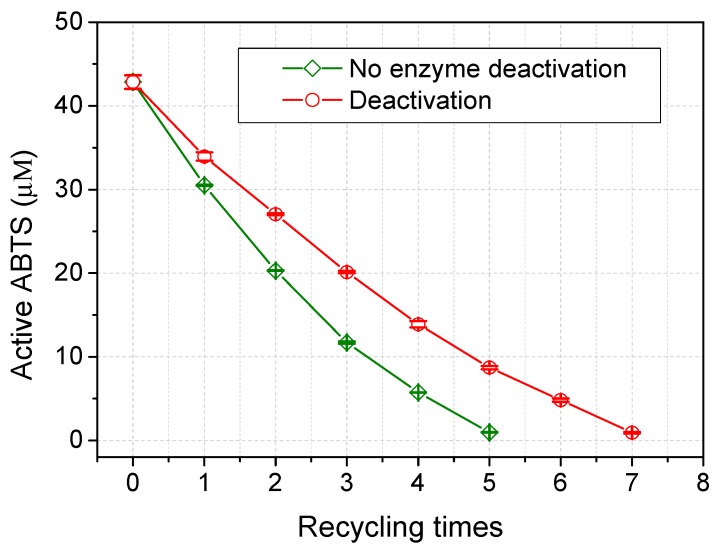
Attenuation of active ABTS radicals with recycling times.

ABTS could be recycled for at least six times under the experimental conditions as shown in Figure 5. Up to 10% active ABTS was successfully retained after six cycles. The recovered ABTS exhibited magenta color with a broad absorption band ranging from 300 to 700 nm which increased with the recycling times. Many authors also reported the red or purple byproduct from laccase-catalyzed ABTS oxidation and deduced it to be azodication (ABTS^2+^). This dication was characterized as an EPR-silent form with UV absorption maxima at 260 and 294 nm [19]. However, Solís-Oba *et al.*, reported contrary findings that ABTS^2+^ is colorless. The red or purple byproduct could be associated to complex formed between the dication and some chemical species present in the solution [20]. To verify these conclusions, ABTS liquor after six recycling processes was ultrafiltered against a Millipore membrane (10 kDa). The filter liquor was colorless while the retention liquor exhibited obvious magenta color, which supports the formation of complex of ABTS^2+^ and laccase.

Deactivation of laccase (by heating) improved the feasibility of ABTS recycling as shown in Figure 5. A probable reason was that ABTS bound to laccase were released from enzyme. Thus, the released ABTS was more readily recovered after mixed with acetone. For control samples (without boiling water treatment), ABTS bound to laccase may be precipitated together in acetone solution, which led to fewer free molecules for recycling. Almost 80% of laccase activity was recovered after solvent evaporation. The residual laccase can re-oxidize recovered ABTS without supplementation. Therefore, the laccase/ABTS active state can be readily controlled by adding or removing acetone in the media, which is particular suitable for process control in biosynthesis.

## 3. Experimental Section

### 3.1. Chemicals and Reagents

Laccase (Novozym 51003, Tianjin, China) activity was determined against substrate ABTS at pH 4.0 [4]. ABTS was purchased from Sigma-Aldrich (St. Louis, MO, USA). The protein concentration was determined by the Bradford method using BSA as a standard. Other chemicals were of analytical grade.

### 3.2. Laccase/ABTS Reaction

Reaction of laccase and ABTS was performed in a quartz cuvette (1 × 1 × 4 cm^3^) at 30 ± 1 °C. The reaction mixture consisted of 50 mM acetate buffer (pH 4.0), 0.005~0.2 mM ABTS and a certain volume percentage of acetone (see text). The reaction was started by adding a proper amount of laccase which was prescribed to oxidize less than 5% of ABTS in 3 min for measuring Michaelis-Menten constants (*K_m_* and *V*_max_). UV-Vis spectrum was determined by using an Agilent 8453 UV-Vis spectrophotometer (Shanghai, China) at a ten-second interval.

### 3.3. ABTS Recovery Procedure

For recovery study, 45 μmol ABTS was converted to blue-green radicals by laccase (100 U) at 30 °C for 1 h. The reaction mixture was boiled for 10 min in order to inactivate laccase. Acetone was next added to the mixture with a final concentration of 80% (*v*/*v*). Acetone was removed by vacuum-rotary evaporation after incubation at 30 °C for 60 min. The faded ABTS was re-oxidized with 100 U of fresh laccase. The re-generated ABTS radicals (ABTS**^·^**^+^) with a representative absorbance peak at 750 nm were measured for calculating the recovery of active ABTS. The recovery process was repeated until no blue-green radicals could be generated.

## 4. Conclusions

ABTS cationic radicals were transformed to the reductive form by acetone or other water-miscible organic solvents. We could recover up to 95% ABTS in 80% (*v*/*v*) acetone in 60 min. The laccase/ABTS active state was readily manipulated by adjusting acetone concentration in the media. In this study, we provided a simple method to recover an effective mediator that is particularly suitable for process control in biosynthesis.
