# Erratum: Liu, H., *et al*. Radical Scavenging by Acetone: A New Perspective to Understand Laccase/ABTS Inactivation and to Recover Redox Mediator. *Molecules* 2015, *20*, 19907–19913

**DOI:** 10.3390/molecules21070957

**Published:** 2016-07-21

**Authors:** Molecules Editorial Office

**Affiliations:** MDPI AG, Klybeckstrasse 64, CH-4057 Basel, Switzerland

The *Molecules* Editorial Office wishes to report the following erratum to this paper [1]. In the paper, Figure 2 is the same as Figure 3. The correct version of Figure 2 is as follows:

We apologize for any inconvenience caused to the readers by this mistake.

## Figures and Tables

**Figure 2:**
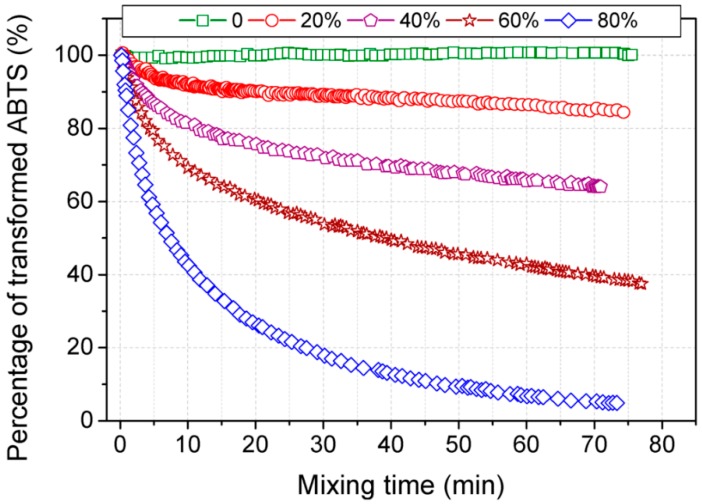
Effects of acetone content on kinetic transformation of ABTS radicals.
